# Detecting low-intake dehydration using bioelectrical impedance analysis in older adults in acute care settings: a systematic review

**DOI:** 10.1186/s12877-022-03589-0

**Published:** 2022-12-12

**Authors:** Saleh Alsanie, Stephen Lim, Stephen A. Wootton

**Affiliations:** 1 School of Human Development and Health, Faculty of Medicine, University of Southampton, Southampton, UK; 2 Department of Clinical Nutrition, College of Applied Health Sciences in Arrass, Qassim University, Buraydah, Saudi Arabia; 3 NIHR Southampton Biomedical Research Centre, University of Southampton and University Hospital Southampton NHS Trust, Southampton, UK; 4 Academic Geriatric Medicine, University of Southampton, Southampton, UK; 5 NIHR ARC Wessex, University of Southampton, Southampton, UK

**Keywords:** Acute care, Bioelectrical impedance analysis, Dehydration, Older adults, Systematic review

## Abstract

**Background:**

Dehydration is a frequent cause of excess morbidity and poor health outcomes, particularly in older adults who have an increased risk of fluid loss due to renal senescence, comorbidities, and polypharmacy. Detecting dehydration is key to instigating treatment to resolve the problem and prevent further adverse consequences; however, current approaches to diagnosis are unreliable and, as a result, under-detection remains a widespread problem. This systematic review sought to explore the value of bioelectrical impedance in detecting low-intake dehydration among older adults admitted to acute care settings.

**Methods:**

A literature search using MEDLINE, EMBASE, CINAHL, Web of Science, and the Cochrane Library was undertaken from inception till May 2022 and led to the eventual evaluation of four studies. Risk of bias was assessed using the Cochrane tool for observational studies; three studies had a high risk of bias, and one had a low risk. Data were extracted using systematic proofs. Due to insufficient reporting, the data were analysed using narrative synthesis.

**Results:**

One study showed that the sensitivity and specificity of bioelectrical impedance in detecting low-intake dehydration varied considerably depending on the total body water percentage threshold used to ascertain dehydration status. Other included studies supported the technique's utility when compared to conventional measures of hydration status.

**Conclusions:**

Given the scarcity of literature and inconsistency between findings, it is not possible to ascertain the value of bioelectrical impedance for detecting low-intake dehydration in older inpatients.

**Supplementary Information:**

The online version contains supplementary material available at 10.1186/s12877-022-03589-0.

## Background

Dehydration is a highly prevalent and burdensome problem that disproportionately affects older hospitalised patients due to their age and comorbidity risk of excess fluid loss and insufficient fluid intake, which results in a net fluid deficit [1]. In physiological terms, dehydration can be defined as a relative reduction in total body water (TBW) volume to less than an individual’s usual volume, leading to impaired renal and haemodynamic functions involved in the regulation of blood pressure and systemic organ perfusion [2]. The prevalence of dehydration among the older population varies by geographic region and patient setting and has been reported to be as high as 39% among nursing home residents and approximately 25% among hospitalised patients [3]. The increased risk of dehydration among older adults has been attributed to the age-related decline in renal function, also known as renal senescence, with glomerular filtration rate decreasing by more than 50% over the ages of 30–80 years [4, 5].

In most cases, dehydration can be avoided through adequate fluid intake, although chronic comorbidities, such as diabetes, renal disease, cognitive impairment, mental health problems, and polypharmacy, increase the risk and are commonly observed among those with recurrent hydration problems [6]. Common causes of excess morbidity and mortality include hypovolaemia, hypotension, electrolyte disturbances, cardiac arrhythmias, delirium, seizures, renal failure, and hypovolaemic shock [7]. In a landmark study based on 10 million hospital records of patients admitted to hospitals in the United States, older adults with diagnosed dehydration observed 30-day mortality of 17% and one-year mortality of 48%, highlighting that dehydration is a significant issue affecting the vulnerable older population [8].

The recent European Society for Clinical Nutrition and Metabolism (ESPEN) guidelines for hydration in the field of geriatrics state that dehydration in older adults is due to either low fluid intake (due to a lack of drinking), excess fluid loss, or a combination of both; practice guidance remains the mainstay for hydration care in this population group worldwide [9, 10]. The ESPEN guidelines specifically define low-intake dehydration as a shortage of body water that leads to reductions in intracellular and extracellular fluid and, subsequently, increased osmolality across both cellular compartments.

Accurate detection of impending dehydration is key to preventing complications, excess morbidity and mortality among predominantly older adults who represent an already at-risk group for poor outcomes during and following hospitalisation. There is no reliable objective method to assess dehydration in clinical practice to both diagnose and confirm the resolution of dehydration. The diagnosis of dehydration has traditionally depended on clinical symptoms and signs such as moisture of mucous membranes and physiological responses to hypovolaemia, including tachycardia and reductions in blood pressure from baseline values in those who are severely dehydrated [11]. In patients with early or minor dehydration, clinical assessment methods are markedly insensitive, and are associated with delays in initiating hydration therapy and increased risk of complications [12]. More objective measures such as urea, creatinine, and plasma osmolality, and the assessment of urine colour, output, and osmolality, as well as body weight are available not not used routinely in practice due to insufficient sensitivity and reliability, inhibiting early diagnosis and treatment [13, 14].

Bioelectrical Impedance Analysis (BIA) is a portable, easy-to-use, inexpensive and non-invasive method, that is accessible at the point of care and can be repeated frequently with minimal consumable costs [15, 16]. It measures whole-body impedance (Z), the opposition of the body to alternating current consisting of two components: resistance (R) and reactance (Xc). Resistance is the decrease in voltage reflecting conductivity through ionic solutions. Reactance is the delay in the flow of current measured as a phase-shift, reflecting dielectric properties, i.e., capacitance, of cell membranes and tissue interfaces. Both measures will alter with changes in hydration status. BIA is not a direct method for the assessment of body composition and its utility relies on the relationship between impedance measures and the fluid and electrolyte status of the body. In the euhydrated state, impedance measures can be used to predict estimates of total body water (as well as intracellular and extracellular fluid water), and in turn, the proportions of fat and lean by applying suitable (i.e. age-, sex- and population- and device-specific) equations for the calculation of body compartments. However, these conditions are frequently violated in sick and hospitalized patients since disturbed hydration or altered distribution of extra- and intra-cellular water are often present. In contrast, the measured values of resistance and reactance, and the derived parameter of Phase Angle, are not affected by the factors that affect the assumptions used in the estimation of body composition, have both excellent accuracy and precision, and may offer an objective measure that can be used to mark differences in hydration status in older people at risk of low-intake dehydration in the clinical setting [16–18].

In summary, low-intake dehydration is a common problem that predominantly affects older patients with intermittent illness and can lead to excess morbidity when undetected and untreated. There are various approaches to the diagnosis of low-intake dehydration, including clinical examination and objective quantitative measures of hydration status such as plasma and urine osmolality and specific gravity, although neither of these, whether used in isolation or combination, are sufficiently accurate to diagnose dehydration. More recently, BIA has emerged as a novel approach to diagnosing low-intake dehydration, although this requires further evaluation.

This systematic review specifically sought to explore the use of BIA for low-intake dehydration among older adults admitted to acute hospital care facilities.

## Methods

A search for literature pertinent to the research question was undertaken using the electronic databases Ovid MEDLINE and EMBASE, CINAHL (EBSCO), Web of Science Core Collection (indexes SCI Expanded, SSCI, A&HCI, CPCI-S, CPCI-SSH, ESCI), and Cochrane Central and CDSR. The search terms, syntaxes, and Boolean operators used for database searching are detailed in Table 1 in accordance with the accepted population, exposure/interest, outcomes, and setting (PE/IOS) framework [19]. Details of the search strategy for each database are included in appendices.

**Table 1:** Search strategy informed by the PE/IOS framework

PE/IOS	Search Terms and Boolean Combinations
Population	‘Geriatrics’ OR ‘aged’ OR ‘aged subject’ OR ‘frail elderly’ OR ‘old* adult*’ OR ‘old* person*’ OR ‘old* people’ OR ‘old* patient*’ OR ‘old* m#n’ OR ‘old* wom#n’ OR ‘old* age’ OR ‘elder*’ OR ‘old* male*’ OR ‘old* female*’ OR ‘old* population*’ OR ‘geriatric*’ OR ‘elderly people’ OR ‘elderly person’ OR ‘ageing’ OR ‘aging’ OR ‘senior citizen*’
Exposure/interest	‘Bioelectrical impedance analysis’ OR ‘bioelectrical’ OR ‘electric impedance’ OR ‘impedance’ OR ‘BIA’ OR ‘reactance’ OR ‘resistance’ OR ‘bioimpedance’ OR ‘bioimpedance analysis’ OR ‘electrical’ OR ‘phase angle’ OR ‘ohmic’ OR ‘capacitance’
Outcomes	‘Hydrat*’ OR ‘dehydrat*’ OR ‘euhydrat*’ OR ‘rehydrat*’ OR ‘body water’ OR ‘body fluid*’ OR ‘hypohydrat*’ OR ‘fluid* balance*’ OR ‘fluid* imbalance*’ OR ‘fluid* measur*’ OR ‘fluid* monitor*’ OR ‘water* volum*’ OR ‘water* intake’ OR ‘water* balance*’ OR ‘water* imbalance*’ OR ‘water* measur*’ OR ‘water* monitor*’ OR ‘fluid* deficit*’ OR ‘fluid* manag*’ OR ‘liquid* manag*’ OR ‘liquid* volum*’ OR ‘liquid* intake’ OR ‘liquid* balance*’ OR ‘liquid* imbalance*’ OR ‘liquid* measur*’ OR ‘liquid* monitor*’
Setting	‘Hospital*’ OR ‘clinical care’ OR ‘acute care’ OR ‘hospitalisation’

*PE/IOS* population, exposure/interest, outcomes, and setting

key: ^*^truncation syntax

A literature search was performed for randomised controlled trials and observational cross-sectional, cohort, and case–control designs. Search results were limited for publications in peer-reviewed journals in English Language, with time limit from inception till May 2022, and publications which reported on each of the PE/IOS components. The inclusion criteria was both male and female older adults (defined as age ≥ 65 years), as this is the usual age threshold to define the population group of older persons who are most affected by dehydration. Such persons had to have low-intake dehydration measured using BIA during the receipt of care within a hospital setting. Studies were not limited by publication date or geographic setting, as it was pertinent to include all relevant evidence. Peer-review was considered necessary to identify and evaluate evidence of sufficient scientific and ethical rigour [20]; details regarding peer-review were either determined from the journal website or from databases indexing the journal. The criteria for publications in English language was necessary to comprehend and collectively analyse the reported outcomes without the need for translation. Studies among children and younger adults were excluded from the review because of the low rate of low-intake dehydration among these population groups. Finally, outcomes regarding the value of BIA for detecting low-intake dehydration had to comprise indices of diagnostic accuracy, such as sensitivity and specificity, as these are widely used among diagnostic accuracy reviews and are amenable to pooled statistical analyses [21]. The inclusion and exclusion criteria are presented in Table 2.

**Table 2:** The inclusion and exclusion criteria used in the review

Study Characteristics (PE/IOS)	Inclusion Criteria	Exclusion Criteria
Research design	Randomised controlled trials and observational studies, including cross-sectional, cohort, and case–control studies	Secondary review research, animal, laboratory-based, and qualitative studies, editorials, letters, case series, and case reports
Publication date	No restriction	-
Language	English	Other languages
Peer-reviewed research	Journals	Articles not subject to peer review
Geographical region	No restriction	-
Study quality	No restriction	-
Population	Older adults aged ≥ 65 years with low-intake dehydration (plasma osmolality ≥ 295 mOsm/kg)	Younger adults aged 18–64 years or children aged < 18 years Older adults with euhydration or plasma osmolality < 295 mOsm/kg
Exposure/interest	Hydration status measured using BIA	-
Outcomes	Diagnostic value, including measures of sensitivity, specificity, total accuracy, and/or positive or negative predictive values	Outcomes irrelevant to the research question
Setting/context	Hospital or other acute healthcare facilities	Community care facilities

*BIA* bioelectrical impedance analysis, *PE/IOS* population, exposure/interest, outcomes, and setting

**Fig. 1:**
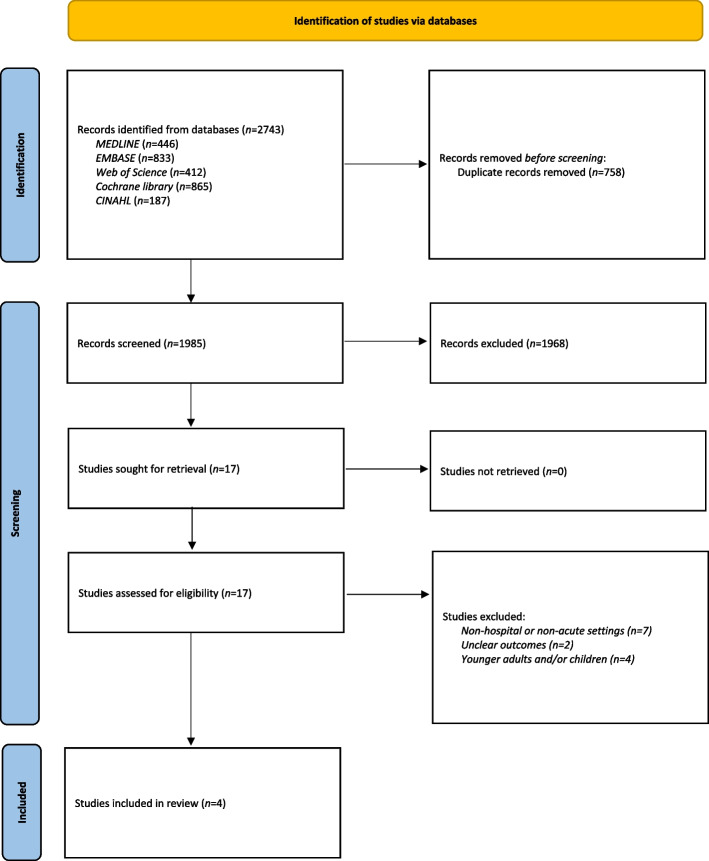
The process by which the studies were selected, depicted using the PRISMA flow diagram [25]. PRISMA: Preferred Reporting Items for Systematic Reviews and Meta-Analyses

Studies were selected using the usual filtering process of title/abstract and full-text screening, with citations managed using Clarivate Analytics® EndNote X9 referencing software [22]. The results of the study selection are presented in the Results section and in the Preferred Reporting Items for Systematic Reviews and Meta-Analyses (PRISMA) flow diagram in Fig. 1 [23, 24].

The data required for critical appraisal and results synthesis were systematically extracted using pre-developed electronic proformas taken from the Cochrane Handbook for Systematic Reviews and adapted to suit the construct of interest [26]. Data extraction was conducted by two reviewers (SA and SL). Any discrepancies were discussed with a third reviewer (SAW) to reach a consensus. Quality assessment of eligible studies was performed using the Cochrane Risk-of-Bias Tool for Randomised Controlled Trials and a modified version of the Cochrane tool for non-randomised studies [27, 28]. The risk of bias for each study was rated in accordance with Cochrane guidelines as either low, high, or unclear; judgements regarding external validity are noted in the discussion section of this report. Data regarding the diagnostic utility of BIA included consideration of pooled meta-analyses, which would have been conducted using the Cochrane Collaborations RevMan® v5.3 software®. However, the outcome data were not amenable to meta-analyses due to the lack of reporting of true positives, true negatives, false positives, and false negatives. As only one study in the review reported diagnostic accuracy indices, a consistent analytical approach in the form of narrative synthesis was undertaken to describe the value of BIA for detecting low-intake dehydration [29].

## Results

### Study selection

Following the search for literature using the defined strategy, a total of 2,743 studies were retrieved. Before screening for titles/abstracts, 758 duplicates were discarded. The remainder 1,985 studies were screened for titles/abstracts, and 1,968 studies which did not fit inclusion criteria or were irrelevant were excluded, leaving 17 articles for full-text review. This final process led to the further exclusion of 13 studies for the following reasons: 1) evaluation of BIA used among older adults in non-hospital or non-acute setting; 2) unclear outcomes regarding the diagnostic value of BIA for low-intake dehydration in older adults; 3) evaluation of BIA used among younger adults and/or children. The remaining four studies met each of the inclusion criteria and were therefore deemed eligible for review.

### Study characteristics

The research designs of the four studies (Table 3) identified for collective review [28,29,30,] comprised two single-centre prospective observational cohort studies [30, 31], a multi-centre prospective cohort study [22] and a randomised non-controlled study [32].

**Table 3:** The selected studies included in the current review

Author and Year	Design	Setting	Participants (sample size)	Bioelectrical Impedance Analysis	Comparators
**Jones, et al. **[30] (2015)	Prospective, observational cohort (single centre)	Australia	Subjects (*n* = 61) admitted to the ICU who received mechanical ventilation and with an expected hospital stay of ≥ 48 h Patients with dehydration had a mean age of 66 years Sex: Female (38%) and male patients (62%)	Bioelectrical impedance vector analysis (Renal EFG BIVA™ Technology; EFG Diagnostic, Belfast, UK) Impedance of 50 kHz Pairs of electrodes were placed on the dorsum of the wrist and the ipsilateral ankle	A comparator was not used
**Kafri, et al. **[31] (2013)	Prospective, observational cohort (single centre)	United Kingdom	Older adults (*n* = 27) admitted to hospital with acute stroke Patients with dehydration had a mean age of 73.5 years Sex: Not reported	Multi-frequency BIA (Maltron BioScan® 920–2; Maltron International, Essex, UK) Impedance of 5 kHz, 50 kHz, and 100 kHz Pairs of electrodes were placed on the talus and the third and fifth digits of the foot and the third and fifth knuckles of the hand and the wrist	Serum osmolality was analysed using freezing point depression (295–300 mOsm/kg) (i.e. impending dehydration), with current dehydration being ≥ 301 mOsm/kg Serum osmolarity (mOsm/L) was calculated from combined concentrations of serum sodium, potassium, glucose, and urea ([2 × Na +] + [2 × K +] + urea + glucose) (i.e. 295–300 mOsm/L [impending dehydration], with dehydration at the time of the study of ≥ 301 mOsm/L)
**Powers, et al. **[22] (2009)	Randomised non-controlled study	United States	Older adults (*n* = 32) admitted to medical and surgical wards Patients with dehydration had a mean age of 77.1 years Sex: Female (63%) and male patients (37%)	Single-frequency BIA (Real-Time RJL Systems® Analyser, Clinton Township, Michigan, USA) Impedance of 50 kHz Pairs of electrodes were placed on the dorsal surfaces of the right hand and foot proximal to the metacarpal, phalangeal, and metatarsal phalangeal joints One additional pair of electrodes were applied at the pisiform bone of the right wrist and between the medial and lateral malleoli of the right ankle	TBW was determined by ^2^H_2_O dilution, and ECW was measured by using sodium bromide (NaBr) dilution The participants provided baseline blood samples To measure TBW, the participants were asked to drink water containing ^2^H_2_O at an amount of 30 mg/kg of body weight. To measure ECW, the participants were asked to drink water containing NaBr at an amount of 70 mg/kg of body weight A second blood sample was obtained 3–4 h after the oral dose. Plasma was separated from the blood samples Isotope ratio mass spectroscopy was used to determine ^2^H_2_O in the plasma. TBW was calculated using the following equation: TBW = [^2^H_2_Odose/(^2^H_2_O 3 h – ^2^H_2_O 0 h)]/1.04 To measure ECW, NaBr dilution was assayed by using a high-performance liquid chromatography anion-exchange method after serum ultrafiltration. The equation used for the ECW calculation was: ECW = [Br Dose/(Br dose 3 h – Br dose 0 h)] × 0.90 × 0.95 ICW was calculated as TBW – ECW
**Ritz **[32] (2001)	Prospective observational cohort (multi-centre)	France	Older adults (*n* = 169) admitted to geriatric wards for acute medical problems across six hospitals Patients with dehydration had a mean age of 81.4 years Sex: Female (64%) and male patients (36%)	Multi-frequency BIA (Analycor-3®; Spengler, Cachan, France) Impedance of 5 kHz, 50 kHz, and 100 kHz, with current of 400 µA Pairs of electrodes were placed on the distal end of the third metacarpal bone and the distal end of the second metatarsal bone. One additional pair of electrodes was applied between the styloid processes of the radius and ulna and between the two malleoli of the ankle	Dilution measurements of deuterated water (H_2_^18^O) for TBW and Br dilution for ECW The patients were considered dehydrated if they had plasma sodium levels of ≥ 142 mmol/L, and they were considered euhydrated if their plasma sodium concentrations were ≤ 135 mmol/L At baseline, overnight fasting (approximately 12 h) was required, and the participants provided plasma and urine samples to determine the natural abundance of H_2_^18^O enrichment and Br concentration An amount of 2% of H_2_^18^O-enriched water (approximately 50 g) was orally administered to the subjects, and 20 g of potassium Br syrup (containing approximately 1 g of Br) was given to half the participants. After an interval of 4–5 h, the plasma and urine samples were collected The following equation was used to calculate ECW after considering the mean Br plasma concentration 4 and 5 h after the dose: ECW = 0.90 × 0.95 × (Br dose) ⁄ [delta(Br plasma)] Br dose = the dose given and delta (Br plasma) = the difference in mean plasma concentration between the administration of the dose and the baseline concentration

^*2*^*H*_*2*_*0* deuterium oxide, *Br* bromide, *EBW* extracellular water, *ICU* intensive care unit, *NaBr* sodium bromide, *TBW* total body water, *H*_*2*_^*18*^*O* water enriched with oxygen‐18

A summary of the findings of the four studies is presented in Table 4. The populations and sample sizes were as follows: older adults (*n* = 61) admitted to the intensive care unit who received mechanical ventilation and had an expected length of stay of ≥ 48 h [30], older adults (n = 27) admitted to hospital with acute stroke [31], older adults (*n* = 32) admitted to medical and surgical wards [22], and older adults (*n* = 169) admitted to geriatric wards for acute medical problems across six hospitals [32].

**Table 4:** A summary of the findings of the four included studies

Author and Year	BIA Equipment/Protocol	BIA data (R [Ohm/m]; Xc [Ohm/m] and Pa [degrees])	Measure of Hydration Status	Descriptive Findings
**Jones, et al. **[30] (2015)	Bioelectrical impedance vector analysis (Renal EFG BIVA™ Technology; EFG Diagnostic, Belfast, UK) Impedance of 50 kHz Pairs of electrodes were placed on the dorsum of the wrist and the ipsilateral ankle The measurements were taken twice daily (in the morning and afternoon) for the first five days of each patient’s stay in the ICU or until ICU discharge, where the patients were positioned horizontally and placed in the supine position for ≥ 2 min	Dehydration (*n* = 14): R = 321.0 Xc = 43.6 Pa = 9.1 Euhydration (*n* = 22): R = 314.0 Xc = 29.6 Pa = 95.5 Overhydration (*n* = 25): R = 224.0 Xc = 16.5 Pa = 3.8	A quantitative estimation was made of TBW volume as a percentage of fat-free body mass Dehydration (TBW of ≤ 72% of fat-free body mass) Euhydration (TBW of 73–74% of fat-free body mass) Overhydration (TBW of ≥ 75% of fat-free body mass)	BIA was used to categorise the patients according to hydration status using TBW volume thresholds of ≤ 72% (signifying dehydration), 73–74% (reflecting normal hydration), and ≥ 75% (indicating overhydration). While no direct measures of diagnostic accuracy were reported given the lack of a comparator group, the results showed that BIA reliably identified dehydration based on positive physiological responses to fluid challenges and maintenance fluid therapy. Dehydration, identified using BIA, was associated with a non-significant increase in the requirement for renal replacement therapy (*p* = 0.800), length of ICU stay (*p* = 0.870), length of hospital stay (*p* = 0.220), admission to intensive care (*p* = 0.890), and the rate of hospital mortality (*p* = 0.550) compared to normally hydrated subjects (all *p* > 0.05)
**Kafri, et al. **[31] (2013)	Multi-frequency BIA (Maltron BioScan® 920–2; Maltron International, Essex, UK) Impedance of 5 kHz, 50 kHz, and 100 kHz Pairs of electrodes were placed on the talus and the third and fifth digits of the foot and on the third and fifth knuckles of the hand and the wrist The participants fasted for at least two hours and were asked to remove any jewellery and to micturate if they wished before the BIA measurements were taken Two consecutive measurements were taken over a couple of seconds within 20 min of the blood samples being taken while the subjects were in the supine position. The recordings were repeated a few minutes later. An average of the two consecutive measurements was calculated. The first data set was used in the event of variation of ≥ 3% Height, weight, gender, age, and ethnicity were entered into the MF-BIA device BIA outputs (the mean of the two readings) were used to calculate TBW (L) and ECW (L) using published equations for older people TBW, ECW, and ICW were calculated as body weight percentages using equations specifically developed for older adults (rather than those already programmed in the device) TBW was estimated using the Vaché equation: TBW = (2.896) + (0.366*height^2^/R100) + (0.137*weight) + (2.485*G) R100 = impedance at 100 Hz, and G = gender, with a value of 1 for men and 0 for women ECW was estimated using the Visser equation: ECW (Women) = (1.7) + (0.2*height^2^)/(R5) + (0.057*weight) ECW (men) = (4.8) + (0.225* height^2^)/(R5)	Not reported	A quantitative estimation was made of (1) TBW volume as a percentage of body weight, (2) ICW as a percentage of TBW, and (3) ECW as a percentage of TBW using MF-BIA (using published equations for older adults)	The accuracy of BIA varied with the threshold of TBW volume congruent with dehydration. The highest sensitivity (100%) was observed for TBW volume of 55% with low corresponding specificity (i.e. of only 14%). The highest specificity (91%) was observed for a TBW volume of 45%; however, the corresponding sensitivity was only 17%. The positive and negative predictive values were 25–33% and 79–100%, respectively. Optimal accuracy with a modest sensitivity and specificity (62–67%) was observed for a TBW volume threshold of 52%
**Powers, et al. **[22] (2009)	Single-frequency BIA (Real-Time RJL Systems® Analyser, Clinton Township, Michigan, USA) Impedance of 50 kHz Pairs of electrodes were placed on the dorsal surfaces of the right hand and foot proximal to the metacarpal, phalangeal, and metatarsal phalangeal joints One additional pair of electrodes was applied at the pisiform bone of the right wrist and between the medial and lateral malleoli of the right ankle The participants were placed in the supine position, with their arms and legs abducted at an angle of 30–45°. Overnight fasting was required	Not reported	R, Xc, Ht, wt, gender, age, and amount of exercise were entered into a software programme called Cyrus TBW and ECW were estimated using the BIA device	The use of BIA was associated with small inter-individual variability in relation to the accurate measurement of TBW volume percentage (4.1%), compared to the reference tests, which suggests that the method feasibly identified dehydration and changes in hydration status. ECW volume, measured using BIA, was not significantly different to that measured using NaBr (*p* = 0.430)
**Ritz **[32] (2001)	Multi-frequency BIA (Analycor-3®; Spengler, Cachan, France) Impedance of 5 kHz, 50 kHz, and 100 kHz, with current of 400 µA Pairs of electrodes were placed on the distal end of the third metacarpal bone and the distal end of the second metatarsal bone. One additional pair of electrodes was applied between the styloid processes of the radius and ulna and between the two malleoli of the ankle The measurements were taken on both sides of the body. Overnight fasting (approximately 12 h) was required. The measurements were taken after resting for at least 30 min and up to five hours post the administration of the H_2_^18^O and Br doses	Not reported	A quantitative estimation was made of TBW and ECW as a percentage of body weight TBW was estimated at 50 kHz and 100 kHz using the following equations: TBW (l) 2.896 0.366 Ht^2^ = + ⁄ I100 + 0.137 wt + 2.485G TBW (l) 3.026 0.358 Ht^2^ = + ⁄ I50 + 0.149 wt + 2.924G ECW was estimated at 5 kHz using the following equations: ECW (Segal,1) = -6.1 + 0.284 Ht^2^/I5 + 0.112 wt ECW (Visser, men,1) = 4.8 + 0.225 Ht^2^/I5 ECW (Visser, women,1) = 1.7 + 0.2 Ht^2^/I5 + 0.057 wt In all the equations, Ht was measured in centimetres, and wt in kilogrammes. I signifies ‘impedance’, and G ‘gender’ (with values of 0 and 1 for women and men, respectively)	Sufficient comparability was observed between BIA and the reference tests in measuring TBW volume; notably, the method was able to discriminate between dehydration and normal hydration based on a TBW volume of 0.25–0.39 L. In this regard, average TBW volume in normally hydrated subjects was 0.69–0.83 L, considerably higher than that in dehydrated subjects

*BIA* bioelectrical impedance analysis, *Br* bromide, *ECW* extracellular water, *Ht* height, *ICU* intensive care unit, *ITW* intracellular water, *MF-BIA* multi-frequency bioelectrical impedance analysis, *NaBr* sodium bromide, *Pa* phase angle, *R* resistance, *TBW* total body water, *wt* weight, *Xc* reactance, *I* impedance

The mean age of subjects across the studies ranged between 63 and 80.1 years; the study of subjects with a mean age of 63 years reported by Jones et al. [30] was included due to the predominance of older adults in the cohort. Patient hydration status was ascertained using the following techniques: bioelectrical impedance vector analysis [30], multi-frequency BIA [22, 31], and single-frequency BIA [32].

BIA outcome measures used to determine hydration status differed between studies and included TBW percentage, intracellular water percentage, extracellular water percentage, and extracellular water:intracellular water ratio. Only one study [31] reported diagnostic accuracy indices, as noted in the meta-analysis and narrative synthesis subsections.

### Quality assessment

As no studies included in the review were randomised controlled trials, the Risk of Bias for Non-Randomised Studies tool was used to inform the risk of bias among the observational studies [33]. A summary of the assessments is provided in Table 5. Overall, three studies were rated as having a low risk of bias [22, 30, 32], whilst the remaining study observed an unclear risk of bias due to uncertainty regarding selection bias and bias related to missing data [31]. Specific insight into the factors leading to such judgements of quality is provided below, in accordance with the recommendations of Mallen, et al. [33].

**Table 5:** Critical appraisal of the quality of the included studies using the Cochrane ROBINS-I tool [34]

Included Studies	Selection Bias	Confounding Bias	Classification of Exposure Bias	Missing Data Bias	Outcome Measurement Bias	Reporting Bias	Overall Risk of Bias
Jones, et al. [50]	L	L	L	L	L	L	L
Kafri, et al. [51]	U	L	L	L	H	H	H
Powers, et al. [53]	L	L	L	L	H	H	H
Ritz [52]	L	L	L	L	H	H	H

^*^U unclear; *H* high, *L* low

Two of the studies [22, 30] recruited subjects using consecutive sampling techniques, which is a credible approach to avoiding selection bias in non-randomised observational studies, given that there is no risk of selectivity in including or excluding participants with characteristics that may skew measured outcomes. Of the two other studies, Powers, et al. [32] utilised random sampling, while the sampling technique was not sufficiently described by Kafri, et al. [31], leading to low and unclear selection bias risk judgements, respectively. As each studied different specific populations the external validity (generalisability of the findings to all older patients) is poor. Jones, et al. [30] restricted subjects to those admitted to the intensive care unit and who were expected to be ventilated for longer than 48 h, while Kafri, et al. [31] included subjects with incident stroke, which co-existed with extensive exclusion criteria, thus impairing external validity to the general older population. Ritz, et al. [22] and Powers, et al. [32] and included older adults admitted to medical and surgical wards for various clinical reasons and with minimal exclusion criteria offering broader generalisability to other older adult populations. Sample size also affected the external validity of most studies [30–32] in this review, with only one study [22] attaining a reasonably sized representative sample (i.e. 169 subjects).

The studies included in this review were judged to have a low risk of confounding bias, as the authors accounted for multiple demographic and clinical factors in the statistical analyses, which were considered important or potential influencers of hydration status. There was also a minimal risk of misclassification bias across all studies in this review, given that evidence-based thresholds were used to categorise subjects into hydration status categories (euhydrated, dehydrated, and over-hydrated). There was a low risk of outcome measurement bias due to the homogenous derivation of hydration status based on calculations of TBW through BIA. The study by Jones, et al. [30] was the only one to denote/report on the raw data measures for BIA, including resistance, reactance, and phase angle, and thus was considered to have a low risk of reporting bias. While the other three studies in this review did not report the raw data measures but used these to derive the predicted values of TBW, the risk of reporting and measurement biases were high. This might have affected the overall accuracy in diagnosing the hydration status of the participants as the raw data measures are independent of regression equations or weight and can be carried out in situations where BIA assumptions are not valid for estimating body fluid compartments.

### Narrative synthesis

In the study conducted by Kafri, et al. [31], the authors determined the diagnostic accuracy of BIA for dehydration by comparing BIA-derived estimates of TBW with measurements of plasma osmolality. The diagnostic accuracy was found to vary markedly depending on which threshold of TBW was used to define dehydration. The highest sensitivity (100%) was observed for a TBW percentage threshold of 55%, although the corresponding specificity was only 14%. The positive and negative predictive values were 25% and 100%, respectively. In contrast, the highest specificity (91%) was observed for the TBW percentage threshold of 45%, although the corresponding sensitivity was only 17%. The positive and negative predictive values were 33% and 79%, respectively. Similar observations were found when diagnostic accuracy was based on derived estimates of intracellular and extracellular water percentages and extracellular to intracellular water ratios, with progressive increases in sensitivity and progressive decreases in specificity when the threshold values increase. The most desirable balance of accuracy was observed at a TBW percentage threshold of 52%, which yielded a modest sensitivity (67%) and specificity (62%).

Powers, et al. [32] found that when compared to estimates of TBW by deuterium dilution. BIA-derived estimates of TBW were comparable with only a small mean difference in TBW percentage (4.1%) with modest inter-individual differences suggesting that the two approaches to estimating TBW were comparable in detecting differences in hydration status; both were far superior to estimates of TBW derived using conventional predictive approaches using anthropometry.

Ritz, et al. [52] also compared BIA-dervied estimates of TBW against estimates of TBW by deuterium dilution in a large multicentre trial in patients with differing degrees of hydration from dehydrated, euhydrated and overhydrated. They found that TBW could be estimated accurately by BIA and whilst there was a small difference in the estimated TBW, this difference was not affected by hydration status and concluded that BIA could be used to moniter changes in fluid balance across a range of hydration disorders.

Finally, in the study reported by Jones, et al. [30], the authors used bioelectrical impedance vector analysis to classify patients into three categories of hydration status using TBW percentage thresholds of ≤ 72% (signifying dehydration), 73–74% (indicating normal hydration), and ≥ 75%(denoting overhydration). They found higher resistance, with lower reactance, and phase angle values in dehydrated than in euhydrated and overhydrated patients; values that differed progressively from states of overhydration to dehydration and reflected the changes in hydration status with therapeutic intervention. The authors also found that dehydration ascertained using bioelectrical impedance vector analysis was associated with non-significant increases in the need for renal replacement therapy and admission to the intensive care unit, intensive care unit and hospital lengths of stay, and the rate of hospital mortality when compared to normally hydrated subjects (all *p* > 0.05).

## Discussion

This systematic review sought to explore the diagnostic utility of BIA for the detection of low-intake dehydration among older adults admitted to acute care facilities. Of the four studies that met the inclusion criteria were identified, only Kafri, et al. [31] reported the diagnostic accuracy of a BIA-derived estimate of TBW against a clinical measure of dehydration (osmolality). The studies by Ritz, et al. [22] and Powers, et al. [32] compared the BIA-dervied estimates of TBW against those derived by deuterium-dilution. Whilst they found some degree of concordance between the different approaches to estimating TBW they did not compare them against other clinical measures. Jones, et al. [30] reported differences in impedance values in those they categorised as dehydrated compared to those who were eu/overhydrated. They adopted a qualitative approach using vector analysis fidning demonstrating changes in the vector with fluid replacement but once again made no comparision against clinical measures. Taken together, the scarcity and quality of published studies and heterogeneity of observations does not permit any firm conclusion as to the diagnostic utility of BIA in the detection of low-intake dehydration in older people in the acute clinical setting.

Some support in using BIA to detect dehydration may be provided by studies in younger adults and children or in the non-acute clinical setings or in the community. Several such studies were revealed by the search strategy but were not included in the final evaluation as they did not meet the inclusion criteria. Of particular interest, Shimizu, et al. [25] showed that resistance measures using BIA could derive thresholds to discriminate dehydration from normal hydration in a cohort of adults from an outpatient department. The authors found that those adults identified as dehydrated using clinical assessment had a higher resistance than those normally hydrated and that resistance correlated well with plasma osmolality and other laboratory biomarker measurements. Similarly, Dal Cin, et al. [35] found that BIA could detect dehydration induced by furosemide therapy in a small series of young adults with normal health. In adults with renal disease, O’Lone et al. [36] demonstrated that multi-frequency bioimpedance spectroscopy in peritoneal dialysis patients was an independent predictor of patient survival whilst Park et al. [37] have demonstrated the cinical usefulness of bioimpedance analysis for assessing volume status in patients receiving maintenance dialysis.

In contrast, Rikkert, et al. [38] showed that the sensitivity of BIA for detecting dehydration among community-dwelling older adults was only 14% when compared to a reference comparator comprising a composite of clinical examination, laboratory tests, and changes in weight. Finally, a recent Cochrane review reported by Hooper, et al. [39] evaluated various measures to detect dehydration in older adults, including BIA, but was primarily based on studies excluded from this review conducted among populations attending non-hospital or non-acute settings. The review concluded that clinical assessment measures of hydration status had greater feasibility, cost-effectiveness, and speed than that derived using BIA.

Based on the limited evidence included in this review, measured impedance values appear to change with altered hydration status but the diagnostic utility of detecting low-intake dehydration in older people in the acute care setting remains unclear. This review has several limitations. Firstly, the literature search comprised an informed series of sources and an extensive series of terms; however, there is a residual risk that one or more studies were precluded from the review. Second, this issue could have been exacerbated by the restriction criteria defined for eligible studies. Third, there were only four studies that met the inclusion criteria which together with marked methodological heterogeneity precluded inter-study comparisons and meta-analysis. Finally, variances in outcomes across the studies could have resulted from a difference in BIA equipment and/or a lack of quality control or calibration of the instruments.

Whilst severe dehydration may be readily identified in the acute setting using conventional clinical assessments, those with less overt or early dehydration may be overlooked, undiagnosed and untreated. Future primary research should explore the usefulness of BIA as an adjunct to aid diagnostic accuracy, especially when there is clinical uncertainty, in older adults in high risk settings such as acute care. Future publications would have greater value if they reported the measured values of resistance, reactance and phase angle in addition to the derived estimates of body water and report how they relate to clinical measures of hydration used in routine care.

## Supplementary Information

**Additional file 1.** 

**Additional file 2.**

**Additional file 3.** 

**Additional file 4.** 

**Additional file 5.**

## Data Availability

The dataset used and analysed during this review are available from the corresponding author on reasonable request.

## Abbreviations

BIA: bioelectrical impedance analysis
ESPEN: European Society for Clinical Nutrition and Metabolism
PRISMA: Preferred Reporting Items for Systematic Reviews and Meta-Analyses
TBW: total body water
