# Supplementary material for: Detecting low-intake dehydration using bioelectrical impedance analysis in older adults in acute care settings: a systematic review
Source: BMC Geriatr. 2022 Dec 12;22:954. doi: 10.1186/s12877-022-03589-0 (PMC9743772; doi:10.1186/s12877-022-03589-0)
Supplement: Supplementary file 1 — Additional file 1. [file 12877_2022_3589_MOESM1_ESM.docx]

**Appendix: CINAHL Search Strategy**

| ID | Search Strategy |
| --- | --- |
|  | (MH "Frail Elderly") OR (MH "Aged") OR (MH "Aged, Hospitalized") OR (MH "Older Adult Care (Saba CCC)") OR "geriatrics or older adults or elderly or aged or older or elder or elderly or over 65" |
|  | "old man" |
|  | "old female" |
|  | (MH "Health Services for the Aged") OR "elder" |
|  | "old* population" |
|  | "elderly people" |
|  | "ageing" |
|  | "aging" |
|  | "senior citizen" |
|  | S1 OR S2 OR S3 OR S4 OR S5 OR S6 OR S7 OR S8 OR S9 |
|  | (MH "Electric Impedance") OR "bioelectrical impedance analysis" |
|  | "bioimpedance analysis" |
|  | "BIA" |
|  | (MH "Electric Capacitance") OR "electrical capacitance" |
|  | "electrical resistance" |
|  | "electrical reactance" |
|  | "electrical reactance" |
|  | "impedance" |
|  | "ohmic" |
|  | (MH "Phase Angle") OR "phase angle" |
|  | S11 OR S12 OR S13 OR S14 OR S15 OR S16 OR S17 OR S18 OR S19 OR S20 |
|  | S10 AND S21 |
|  | (MH "Hydration Status") OR (MH "Hydration (Iowa NOC)") OR "hydration" |
|  | (MH "Dehydration") OR "dehydration" OR (MH "Fluid Volume Deficit (NANDA)") OR (MH "Fluid Volume Deficit (Saba CCC)") OR (MH "Skin Turgor") |
|  | euhydration" |
|  | "hypohydration" |
|  | (MH "Fluid-Electrolyte Balance") OR (MH "Fluid Balance (Iowa NOC)") OR (MH "Fluid Intake-Output Measures") OR (MH "Fluid Therapy") OR (MH "Body Fluids") OR "fluid balance" OR (MH "Risk for Fluid Volume Deficit (NANDA)") |
|  | (MH "Fluid-Electrolyte Balance") OR (MH "FluidElectrolyte Imbalance") OR (MH "Water") OR (MH "Electrolytes") OR "water-electrolyte balance" |
|  | "fluid imbalance" |
|  | "water-electrolyte imbalance" |
|  | "fluid management" |
|  | "water management" |
|  | "liquid management" |
|  | (MH "Fluid Monitoring") OR "fluid monitor*" |
|  | "water monitor*" |
|  | "liquid monitor*" |
|  | "liquid monitor*" |
|  | "fluid deficit" |
|  | "water intake" |
|  | "liquid volum*" |
|  | "liquid balance" |
|  | "liquid imbalance" |
|  | "liquid imbalance" |
|  | "body water" |
|  | (MH "Rehydration Solutions") OR (MH "Oral Rehydration Therapy") OR "rehydration" |
|  | S23 OR S24 OR S25 OR S26 OR S27 OR S28 OR S29 OR S30 OR S31 OR S32 OR S33 OR S34 OR S35 OR S36 OR S37 OR S38 OR S39 OR S40 OR S41 OR S42 OR S43 OR S44 OR S45 |
|  | S22 AND S46 |
|  | (MH "Acute Care") OR "acute care" OR (MH "Acute Care Nurse Practitioners") |
|  | (MH "Acute Care") OR "acute care" OR (MH "Acute Care Nurse Practitioners") |
|  | "clinical care" OR (MH "National Institute for Health and Care Excellence") |
|  | (MH "Hospitalization") OR "hospitalisation" OR (MH "Day Care") |
|  | (MH "Hospitals") OR "hospitals" OR (MH "Hospitals, Public") OR (MH "Hospitals, Private") OR (MH "Hospitals, Special") OR (MH "Hospitals, Urban") |
|  | S48 OR S49 OR S50 OR S51 OR S52 |
|  | S47 AND S53 |
|  | S47 AND S53-limit applied Narrow by Language-English |
