# Supplementary material for: Detecting low-intake dehydration using bioelectrical impedance analysis in older adults in acute care settings: a systematic review
Source: BMC Geriatr. 2022 Dec 12;22:954. doi: 10.1186/s12877-022-03589-0 (PMC9743772; doi:10.1186/s12877-022-03589-0)
Supplement: Supplementary file 2 — Additional file 2. [file 12877_2022_3589_MOESM2_ESM.docx]

**Appendix: Web of Science Core Collection Search Strategy**

| ID | Search Strategy |
| --- | --- |
| 1. 1 | ALL=(Geriatrics OR aged OR aged subject OR frail elderly OR old* adult* OR old* person* OR old* people OR old* patient* OR old* m#n OR old* wom#n OR old* age OR elder* OR old* male* OR old* female* OR old* population* OR geriatric* OR elderly people OR elderly person OR ageing OR aging OR senior citizen*) |
| 1. 2 | ALL=(Bioelectrical impedance analysis OR bioelectrical OR electric impedance OR impedance OR BIA OR reactance OR resistance OR bioimpedance OR bioimpedance analysis OR electrical OR phase angle OR ohmic OR capacitance) |
| 1. 3 | ALL=(Hydrat* OR dehydrat* OR euhydrat* OR rehydrat* OR body water OR body fluid* OR fluid* balance* OR fluid* imbalance* OR fluid* measur* OR fluid* monitor* OR fluid* deficit* OR fluid* manag*) |
| 1. 4 | ALL=(Hospital* OR clinical care OR acute care OR hospitalisation OR hospitalization) |
| 1. 5 | 4 AND 3 AND 2 AND 1 |
| 1. 6 | 5 *AND* **LANGUAGE:** (ENGLISH) |
