# Supplementary material for: Detecting low-intake dehydration using bioelectrical impedance analysis in older adults in acute care settings: a systematic review
Source: BMC Geriatr. 2022 Dec 12;22:954. doi: 10.1186/s12877-022-03589-0 (PMC9743772; doi:10.1186/s12877-022-03589-0)
Supplement: Supplementary file 4 — Additional file 4. [file 12877_2022_3589_MOESM4_ESM.docx]

**Appendix: Ovid MEDLINE Search Strategy**

| ID | Search |
| --- | --- |
|  | geriatrics.mp. or Geriatrics/ or Aged/ |
|  | aged subject.mp. |
|  | frail elderly.mp. or Frail Elderly/ |
|  | old* adult*.mp. |
|  | *Female/ or old* person*.mp. or *Male/ |
|  | old* m#n.mp. |
|  | old* population*.mp. |
|  | elderly people.mp. |
|  | elderly population.mp. |
|  | Aging/ or ageing.mp. |
|  | senior citizen.mp. |
|  | 1 or 2 or 3 or 4 or 5 or 6 or 7 or 8 or 9 or 10 or 11 |
|  | Electric Impedance/ or bioelectrical impedance analysis.mp. |
|  | bioimpedance.mp. |
|  | Electric Capacitance/ or capacitance.mp. |
|  | BIA.mp. |
|  | electrical resistance.mp. or Electric Impedance/ |
|  | bioimpedance analysis.mp. |
|  | phase angle.mp. |
|  | Electric Conductivity/ or ohmic.mp. |
|  | reactance.mp. |
|  | 13 or 14 or 15 or 16 or 17 or 18 or 19 or 20 or 21 |
|  | 12 and 22 |
|  | Fluid Therapy/ or hydration.mp. or Dehydration/ or Water/ |
|  | euhydration.mp. |
|  | hypohydration.mp. |
|  | fluid balance.mp. or Water-Electrolyte Balance/ |
|  | Water-Electrolyte Imbalance/ or fluid imbalance.mp. |
|  | fluid measurement.mp. |
|  | fluid management.mp. |
|  | water volume.mp. or Body Water/ |
|  | fluid deficit.mp. |
|  | liquid management.mp. |
|  | liquid volume.mp. |
|  | water intake.mp. or Drinking/ |
|  | liquid intake.mp. |
|  | liquid balance.mp. |
|  | liquid imbalance.mp. |
|  | liquid monitor*.mp. |
|  | 24 or 25 or 26 or 27 or 28 or 29 or 30 or 31 or 32 or 33 or 34 or 35 or 36 or 37 or 38 or 39 |
|  | 23 and 40 |
|  | hospital.mp. or Hospitals/ |
|  | acute care.mp. |
|  | clinical care.mp. |
|  | hospitalisation.mp. or Hospitalization/ |
|  | 42 or 43 or 44 or 45 |
|  | 41 and 46 |
|  | 12 and 22 and 40 and 46 |
|  | limit 48 to (english language) |
