# Supplementary material for: Detecting low-intake dehydration using bioelectrical impedance analysis in older adults in acute care settings: a systematic review
Source: BMC Geriatr. 2022 Dec 12;22:954. doi: 10.1186/s12877-022-03589-0 (PMC9743772; doi:10.1186/s12877-022-03589-0)
Supplement: Supplementary file 5 — Additional file 5. [file 12877_2022_3589_MOESM5_ESM.docx]

**Appendix: Cochrane Search Strategy**

| ID | Search |
| --- | --- |
|  | ("old age") |
|  | ("old aged") |
|  | ("elderly") |
|  | ("frailness") |
|  | ("aging-related") |
|  | old* adult* |
|  | old* person* |
|  | old* people* |
|  | old* patient* |
|  | old* m#n |
|  | old* wom#n |
|  | old* male* |
|  | old* female* |
|  | geriatric |
|  | senior citizen |
|  | #1 OR #2 OR #3 OR #4 OR #5 OR #6 OR #7 OR #8 OR #9 OR #10 OR #11 OR #12 OR #13 OR #14 OR #15 |
|  | ("bioelectrical impedance analyses") |
|  | ("bioelectrical impedance analysis") |
|  | bioelectrical |
|  | electrical impedance |
|  | electrical |
|  | ("reactance") |
|  | ("ohmic resistance") |
|  | ("capacitance") |
|  | ("phase angle") |
|  | ("bioimpedance") |
|  | ("bioimpedance analyses") |
|  | ("bioimpedance analysis") |
|  | resistance |
|  | #17 OR #18 #19 OR #20 OR #21 OR #22 OR #23 OR #24 OR #25 OR #26 OR #27 OR #28 OR #29 |
|  | #16 AND #30 |
|  | ("hydration") |
|  | ("dehydration") |
|  | ("euhydration") |
|  | ("euhydrate") |
|  | ("dehydrate") |
|  | ("hydrate") |
|  | ("hypohydrate") |
|  | ("hypohydration") |
|  | ("fluid balance") |
|  | ("fluid intake") |
|  | ("water balance") |
|  | ("body water") |
|  | ("body fluid") |
|  | fluid* monitor* |
|  | water* volum* |
|  | water* intake |
|  | water* imbalance* |
|  | water* measur* |
|  | water* monitor* |
|  | fluid* deficit* |
|  | fluid* manag* |
|  | liquid* manag* |
|  | liquid* volum* |
|  | liquid* intake |
|  | liquid* balance* |
|  | liquid* imbalance* |
|  | liquid* measur* |
|  | liquid* monitor* |
|  | #32 OR #33 OR #34 OR #35 OR #36 OR #37 OR #38 OR #39 OR #40 OR #41 OR #42 OR #43 OR #44 OR #45 OR #46 OR #47 OR #48 OR #49 OR #50 OR #51 OR #52 OR #53 OR #54 OR #55 OR #56 OR #57 OR #58 OR #59 |
|  | #31 AND #60 |
|  | (hospital) |
|  | ("acute care center") |
|  | ("hospitalisation") |
|  | clinical care |
|  | acute care |
|  | #62 OR #63 OR #64 OR #65 OR #66 |
|  | #61 AND #67 |
